# Early Environmental Exposures and Intracellular Th1/Th2 Cytokine Profiles in 24-Month-Old Children Living in an Agricultural Area

**DOI:** 10.1289/ehp.9306

**Published:** 2006-08-17

**Authors:** Paurene Duramad, Kim Harley, Michael Lipsett, Asa Bradman, Brenda Eskenazi, Nina T. Holland, Ira B. Tager

**Affiliations:** 1 Center for Children’s Environmental Health, School of Public Health, University of California, Berkeley, California, USA; 2 Department of Epidemiology and Biostatistics, School of Medicine, University of California, San Francisco, California, USA

**Keywords:** allergen, breast-feeding, children, endotoxin, flow cytometry, interferon-γ, interleukin-4, organophosphate, pesticide, T-helper cytokines

## Abstract

**Background:**

Children who reside in agricultural settings are potentially exposed to higher levels of organophosphate (OP) pesticides, endotoxin, and allergens than their urban counterparts. Endotoxin and allergens stimulate maturation of the immune response in early childhood, but little is known about the effect of exposures to OPs or to the three combined.

**Objectives:**

In this study, we investigated the relationships between these exposures and T-helper 1 (Th1) and T-helper 2 (Th2) cytokines, biomarkers of allergic asthma, in the subjects of CHAMA-COS (Center for the Health Assessment of Mothers and Children of Salinas), a longitudinal birth cohort in Salinas Valley, California. Exposures were ascertained by interviewer-administered questionnaires and by home visits, and clinical diagnoses were abstracted from medical records. Blood samples were collected at 12 and 24 months of age and analyzed for Th1/Th2 status by flow cytometric detection of intracellular interferon-γ/interleukin-4 cytokine expression.

**Findings:**

Mean Th2 levels were significantly higher in children with doctor-diagnosed asthma and children with wheezing at 2 years of age. In a multiple linear regression model, exclusive breast-feeding at 1 month and pet ownership were associated with 35.3% (*p* < 0.01) and 34.5% (*p* = 0.01) increases in Th1, respectively. Maternal agricultural work and presence of gas stove in the home were associated with a 25.9% increase (*p* = 0.04) and 46.5% increase (*p* < 0.01) in Th2, respectively.

**Conclusions:**

Asthma and wheeze outcomes in children at 24 months of age are associated with elevated Th2 status in children at an early age. Our data further suggest that early exposures to an agricultural environment, breast-feeding, pets, and gas stoves affect the development of children’s Th1/Th2 immune response.

Pesticides are ubiquitous in the environment as a result of widespread agricultural and domestic use. Exposure to organophosphate (OP) pesticides has been linked in a small number of epidemiologic studies to asthma or asthma-related symptoms. In a study of pesticide applicators in Iowa and North Carolina, OP exposure in the preceding year was related to wheeze (Hoppin et al. 2002). Respiratory, asthma-like symptoms were associated with exposures to OPs in occupational and environmental settings among villagers in rural China (Zhang et al. 2002). Another recent study in Southern California of early-life risk factors for asthma reported that those children exposed to herbicides and pesticides in the first year of life had significantly elevated risks of developing asthma (Salam et al. 2004). Other studies (Ernst 2002; Kheradmand et al. 2002; Landau 2001; Peden 2000) have suggested a relationship between pesticide exposure and exacerbation and/or onset of childhood asthma, but a causal role remains to be established.

Children of migrant farmworkers who live in rural agricultural communities may be exposed to higher levels of pesticides than other children because of their proximity to fields and potential “take-home” exposures from their parents (Bradman et al. 2005; Eskenazi et al. 2004; Fenske et al. 2000; Harnly et al. 2005; Lambert et al. 2005). In addition to pesticides, children who reside in agricultural communities are also exposed to bacterial endotoxin and allergens (Braun-Fahrlander et al. 2002; Gereda et al. 2000). Exposure to endotoxin during early childhood is associated with decreased occurrence of hay fever, atopic asthma, and atopic sensitization in children (Braun-Fahrlander et al. 2002; Gehring et al. 2002; von Mutius et al. 2000), whereas early exposure to allergens, such as house dust mite *Dermatophagoides pteronyssinus* (Der p 1), is considered a risk factor for development of asthma in children (Ohshima et al. 2002; Peat et al. 1996; Sporik et al. 1990).

Asthma is characterized by chronic inflammation in the airways and the presence of a predominance of CD4^+^ T-helper 2 (Th2) cells that secrete interleukin (IL)-4, IL-5, and IL-13 cytokines (Romagnani 1994). Th2 cells contribute to the immunopathogenesis of asthma by recruiting eosinophils and mast cells to the airways (de Vries et al. 2000; Kuo et al. 2001; Romagnani 1994) and by inducing B-cells to produce immunoglobulin E antibodies (Yssel et al. 1998). Conversely, T-helper 1 (Th1) cells that secrete interferon (IFN)-γ are thought to protect against the development of asthma by regulating Th2 cytokine production, although a mixed Th1/Th2 pattern has been reported (Heaton et al. 2005). Allergic and asthmatic subjects are more likely to have elevated levels of the Th2 cytokines IL-4 and IL-5, and reduced levels of the Th1 cytokines IFN-γ and tumor necrosis factor (TNF)-β (Cohn et al. 2004; Romagnani 1994; Sanchez-Guerrero et al. 1997). However, increased levels of IFN-γ also have been reported in cases of severe asthma that could involve CD8^+^ T cells (Brown et al. 2003; Magnan et al. 2000).

The rapid uptake of Th1/Th2 cytokines by nearby immune cells requires that blood samples be analyzed soon after collection to account for cytokine absorption kinetics (Pala et al. 2000). This may be difficult in epidemiologic studies where samples are collected in the field and then transported to a laboratory for analysis. Therefore, to study the associations between Th1/Th2 cytokines and environmental exposures and the occurrence of asthma in children, we optimized a flow cytometry-based method of intracellular cytokine detection to analyze transported samples with minimal blood volumes (Duramad et al. 2004). IFN-γ and IL-4 are signature cytokines of Th1 and Th2 cells, respectively (Pala et al. 2000). Because only memory cells would be able to synthesize IFN-γ and IL-4 within the 4-hr stimulus period used in this study, this method allowed us to identify the cellular sources of the cytokines of interest. The aims of the present study were threefold: *a*) to use this method to characterize Th1 and Th2 cytokine profiles in a birth cohort of 12-and 24-month-old children residing in an agricultural community; *b*) to evaluate associations between early environmental exposures in the first year of life and children’s cytokine profiles at 12 and 24 months of age; and *c*) to investigate the associations of these early-life cytokine profiles with the diagnosis of childhood asthma.

## Materials and Methods

### Participants and recruitment

The CHAMA-COS (Center for the Health Assessment of Mothers and Children of Salinas) project, a component of the Center for Children’s Environmental Health Research at the University of California, Berkeley, is a longitudinal birth cohort study of the effects of pesticides and other environmental exposures on the health of pregnant women and their children who live in the Salinas Valley, Monterey County, California (Eskenazi et al. 2003). Several hundred thousand pounds of OP and pyrethroid pesticides are applied annually in this agricultural area (California Environmental Protection Agency 2002).

Pregnant women who entered prenatal care at the county hospital or one of five community clinics were screened for eligibility between October 1999 and October 2000. These facilities serve a low-income, largely Mexican farmworker population. Eligible women were ≥ 18 years of age, < 20 weeks gestation at enrollment, spoke English or Spanish, were eligible for government-subsidized health insurance for low-income individuals (Medicaid), and planned to deliver at the county hospital. A total of 601 women were enrolled, of whom 538 were followed through the delivery of a child. Study protocols were approved by the University of California, Berkeley, human subject review committee in compliance with all applicable requirements. Written informed consent was provided by all subjects.

### Interviews and home inspections

Women were interviewed six times: twice during pregnancy, once shortly after delivery, and when the children were approximately 6, 12, and 24 months of age. All interviews were conducted in English or Spanish by bilingual, bicultural interviewers with standardized questionnaires. Home inspections to assess environmental exposures in the home were conducted once during pregnancy and again at 6, 12, and 24 months. Demographic information was obtained at the initial interview and included the mother’s age, education level, country of birth, and length of time residing in the United States. Information on agricultural work for the mother, father, and other household members, home pesticide use, and exposure to cigarette smoke was obtained at each interview. Each postnatal interview included questions about breast-feeding, child care attendance, and pet ownership (dogs, cats, birds, others). Signs of mold, cockroaches, and rodents in the home were assessed by a trained staff member during home inspections.

Medical records from birth through 24 months of age were abstracted by a registered nurse to determine whether the child had ever been diagnosed with asthma, eczema, bronchitis, bronchiolitis, or pneumonia. A clinician’s diagnosis on the medical records was taken as presence of disease. Because a diagnostic label of asthma versus reactive airway disease reflects a given physician’s judgment and diagnostic preferences, we considered three separate diagnoses of reactive airway disease, all separated by at least 1 month in time, as equivalent to a diagnosis of asthma. Maternal report of asthma symptoms, such as wheezing when the child did not have a cold, was also gathered at each interview.

### Blood collection and sample size

Blood samples were collected from CHAMACOS children at approximately 12 and 24 months of age [median for 12-month collections: 11.9 (range, 11.1–19.1 months) and median for 24-month collections: 23.9 months (range, 21.7–29.0 months)]. Pilot testing for the collection and measurement of Th1/Th2 cytokines was conducted on a set of 36 samples obtained from 12-month-old children who were evaluated between January and March 2002. Analysis of Th1/Th2 cytokine profiles of 24-month-old children was conducted on 239 blood samples collected between January 2002 and June 2003. At 24 months of age, 412 children remained in the study and 335 (81%) provided blood samples. These samples were analyzed for Th1/Th2 if the analysis could be conducted within 48 hr of collection (some samples were collected on weekends or at remote locations and could not be mailed to the laboratory within 1 day of collection) and if the blood sample volume was > 1.0 mL (other assays were assigned priority, based on the study protocol). Th1/Th2 data were obtained for both 12-month-old (*n* = 36) and 24-month-old subjects (*n* = 239), with 22 children having measurements at both time points.

### Intracellular Th1/Th2 cytokine analysis by flow cytometry

Flow cytometric detection of intracellular Th1 and Th2 cytokines in pediatric whole blood has been described previously and validated for use in epidemiologic studies (Duramad et al. 2004). Briefly, 500 μL whole blood was activated for 4 hr with phorbol 12-myristate 13-acetate (PMA) (Sigma, St. Louis, MO) and ionomycin (Sigma). CD4^+^ T-helper cells were detected with Peridinin-chlorophyll-protein (PerCP) labeled antibody (Becton Dickinson, San Jose, CA), and Th1 or Th2 cells were detected with monoclonal antibodies specific for Th1 [IFN-γ/fluorescein isothiocyanate (FITC); Becton Dickinson] or Th2 (IL-4/phycoerythrin; Becton Dickinson), respectively.

**Figure 1:**
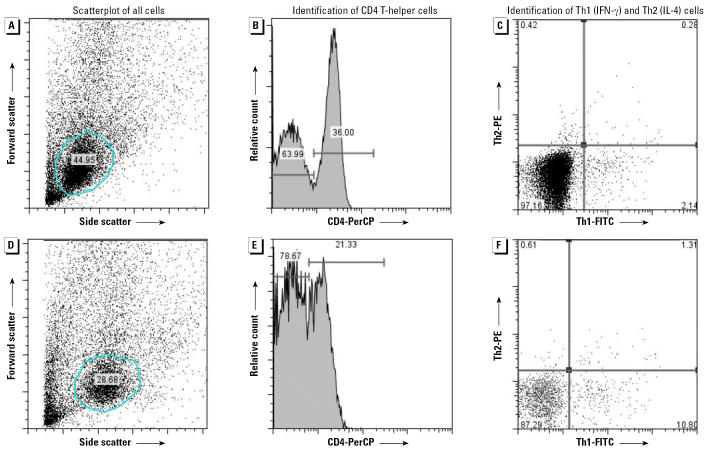
Flow cytometric detection of Th1 (IFN-γ) and Th2 (IL-4) cytokines in whole blood samples collected from children: example of data points included (*A–C*) or excluded (*D–F*) from analysis.

An example of the Th1/Th2 data generated by flow cytometry for each subject is presented in Figure 1. In the scatterplot of all cells (Figure 1A) a circular gate was placed around the lymphocyte population, based on size (FS; forward scatter) and granularity (SS; side scatter). Next, only the CD4^+^ population (right peak, Figure 1B) was examined for expression of IFN-γ and IL-4 (Figure 1C). Cells that stained positive for IFN-γ only (lower right quadrant) were classified as Th1 cells, and those that stained positive for IL-4 only (upper left quadrant) were classified as Th2 cells (Figure 1C).

The percentage of CD4 cells was calculated as the number of CD4 cells divided by total lymphocytes. The percentage of Th1 cells was calculated as the number of IFN-γ positive cells divided by total CD4 cells and the percentage of Th2 was determined as the number of IL-4^+^ cells divided by total CD4 cells. A subject’s data were excluded if the intracellular staining was suboptimal and failed to meet one of the following criteria: *a*) the lymphocyte population was clearly depicted in the scatterplot; *b*) the CD4–PerCP labeling density plot had a clear separation between the negative (left peak) and positive (right peak) CD4 population; and *c*) at least 5,000 CD4^+^ T-helper lymphocytes were counted. For example, the subject depicted in Figure 1D–F was excluded due to suboptimal CD4 staining: There was no clear separation between the negative and positive CD4 populations (Figure 1E). Less than 5% of subjects were excluded due to one or more of these criteria.

### Data analysis

We examined distributions of Th1, Th2, and CD4 graphically and with summary statistics. Differences in levels of Th1, Th2, and CD4 between 12 and 24 months of age were compared with two-tailed *t*-tests and, for those evaluated at both time-points, paired *t*-tests. Because blood draws were not always conducted at exactly 12 or 24 months, we also examined the association of age at blood draw and Th1, Th2, and CD4 status using longitudinal marginal regression models that allowed for analysis of repeated measures. Estimates were obtained using generalized estimating equations with exchangeable correlation and robust standard errors. Subsequent analyses focused only on 24-month levels of Th1, Th2, and CD4. Because Th1 and Th2 had right-skewed distributions, these variables were log-transformed for analysis. CD4 levels were normally distributed and were not transformed. Bivariate analyses using two-tailed unpaired *t*-tests compared the 24-month levels of Th1, Th2, and CD4 among children with either a doctor’s diagnosis of asthma, eczema, bronchitis/bronchiolitis and pneumonia, or maternal report of wheezing without a cold, at 24 months. Bivariate analyses also compared 24-month levels of Th1, Th2, and CD4 according to various environmental exposures with two-tailed *t*-tests and analysis of variance with post hoc tests using Bonferroni adjustment.

We examined Th1 and Th2 cytokine levels, at 24 months, by exposure to pesticides (e.g., parental occupation in agriculture, use of pesticides in home), allergens (e.g., presence of pets, mold, cockroaches, or rodents in home), endotoxin (e.g., parental occupation in agriculture, pets in home) between birth and 12 months of age, as well as other early-life exposures that have been associated with asthma risk in other studies, such as breast-feeding, exposure to cigarette smoke, and gas stove use (Kheradmand et al. 2002; Kull et al. 2004). We focused on exposures during the first year of life, because exposures in this time period have previously been associated with risks for asthma and other immune-related disorders (Bjorksten 1999; Bjorksten et al. 2001; Holladay and Smialowicz 2000; Holsapple et al. 2004; Holt 1998).

We categorized maternal work status into agricultural fieldwork (such as harvesting, picking, thinning, or weeding), other agricultural work (away from the fields, such as packing shed or nursery work), and nonagricultural work/not employed. If a woman performed both fieldwork and other agricultural work, she was classified as doing fieldwork. For fathers and other household members, work status was categorized only into agricultural work and nonagricultural work; maternal work status was also collapsed into these two categories for some analyses. Exclusive breast-feeding was defined as receiving only breast milk, with no supplemental formula, milk or solid foods. Breast-feeding status was categorized as exclusive breast-feeding, partial breast-feeding, or no breast-feeding at 1 month of age. A similar breast-feeding variable was created for 3 months of age. Household income was considered as a covariate and was defined as above or below the federal poverty line, based on the number of people in the household (U.S. Census Bureau 2000).

We constructed separate multiple linear regression models for Th1, Th2, and CD4. Coefficients for the log-transformed measures of Th1 and Th2 were converted back to the natural scale with the equation 100 × (e^β^−1) and are interpreted as the percent change in the outcome associated with a 1-unit change in the independent variable. Initial models included all exposure variables; backwards, stepwise elimination then was used to remove all variables with *p*-values > 0.2. Regression models were repeated using the number of Th1 or Th2 cells (rather than the percentage) as the dependent variable and including CD4 level as an independent variable to confirm that observed associations were due to actual changes in Th1 and Th2 levels and not to changes in CD4 levels that altered the cell ratios. Statistical analyses were performed with STATA version 8.0 (StataCorp., College Station, TX).

## Results

### Th1, Th2, and CD4 status in children at 12 and 24 months of age

**Figure 2:**
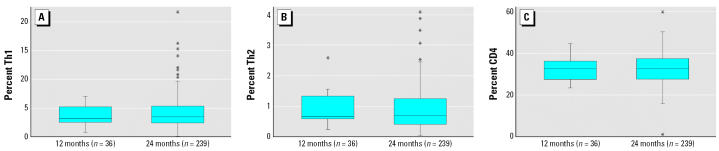
Th1 (*A*), Th2 (*B*), and CD4 (*C*) levels in CHAMACOS children at 12 months (*n* = 36) and 24 months (*n* = 239) of age.

Distributions of Th1, Th2, and CD4, on the arithmetic scale, for children at 12 months (*n* = 36) and 24 months of age (*n* = 239) are presented in Figure 2. The means for the 12-month samples were: Th1 [geometric mean = 3.2%; 95% confidence interval (CI), 2.7–3.9%], Th2 (geometric mean = 0.8%; 95% CI, 0.7–1.0%), and CD4 (arithmetic mean = 32.7%; 95% CI, 30.2–35.3%); and means for 24-month-old sample were: Th1 (geometric mean = 2.9%; 95% CI, 2.6–3.3%), Th2 (geometric mean = 0.7%; 95% CI, 0.6–0.8%), and CD4 (arithmetic mean = 32.9%; 95% CI, 31.9–33.8%). Th1, Th2, and CD4 were not significantly different at 12 and 24 months of age, using both paired *t*-tests of the 22 children with data at both time points and unpaired *t*-tests of the children with independent measurements, and no association was seen between these outcomes and age at blood draw in the longitudinal regression models.

### Characteristics of the CHAMACOS study population

Table 1 describes the sociodemographic characteristics of the subset of the CHAMACOS cohort population whose children had Th1/Th2 cytokine analysis performed at 24 months of age (*n* = 239). Approximately 31% of the women had worked in the fields during pregnancy, and another 8% had worked at other jobs in agriculture (e.g., packing shed, nursery, and greenhouse work). Sixty-three percent of the husbands worked in agriculture, and 60% of mothers reported that pesticides were used in the home. Signs of cockroaches and rodents were seen during inspection of 68% and 43% of the homes, respectively. Children with (*n* = 239) and without (*n* = 175) Th1/Th2 data were similar on most demographic variables, although children with Th1/Th2 data were more likely to have fathers working in agriculture (*p* = 0.03) and to be living below the poverty level (*p* = 0.08). Nearly 10% of the children had been diagnosed with asthma and 7% had been diagnosed with eczema by 24 months of age. Forty-five percent had been diagnosed with bronchitis/bronchiolitis and 29% with pneumonia.

### Associations of Th1, Th2, and CD4 with health outcomes and environmental exposures

Table 2 shows the bivariate associations of Th1, Th2, and CD4 in children at 24 months of age with medical conditions at 24 months, and with exposures between birth and 12 months of age. Children who were diagnosed with asthma had significantly higher Th2 (1.0%; 95% CI, 0.7–1.2%) than those without asthma (0.7%; 95 % CI, 0.6–0.7%; *p* < 0.05). When asthma and reactive airway disease diagnoses were analyzed separately, higher levels of Th2 remained significantly associated with asthma but not with reactive airway disease (data not shown). Similarly, children with maternal report of wheezing without a cold at 24 months had significantly higher Th2 (1.2%, 95% CI, 0.8–1.8%) than children without this condition (0.7%; 95% CI, 0.6–0.8%). No associations were seen between Th1 or CD4 and asthma or wheezing. No associations were found between any of the cytokines and eczema, bronchitis, or pneumonia.

Children who were breast-fed exclusively (i.e., had received no formula, cow’s milk, or solid foods) at 1 month of age had significantly higher Th1 levels (3.3%; 95% CI, 2.9–3.9%) than those who were not breast-fed at all (2.3%; 95% CI, 1.4–3.7%; *p* = 0.03). Although Th1 levels were still elevated in children who were exclusively breast-fed at 3 months (vs. not exclusively breast-fed) and those who had any breast-feeding at 6 and 12 months (vs. none), these findings were not statistically significant (data not shown). Higher levels of Th1 were seen among children who had pets (3.5%; 95% CI, 3.0–4.1%) than children whose homes did not have pets (2.7%; 95% CI, 2.3–3.2%; *p* = 0.04). A similar pattern of increased Th1 was observed in children with indoor pets (3.6%; 95% CI, 2.7–4.6%) compared with those without (2.7%; 95% CI, 2.3–3.2%; *p* = 0.25).

Children who lived with agricultural workers had higher levels of Th2 (0.8%; 95% CI, 0.7–0.9%) than children who did not (0.6%; 95% CI, 0.5–0.7%; *p* = 0.02). Specifically, children of women who worked in the fields had significantly higher Th2 (0.9%; 95% CI, 0.7–1.0%) than children of mothers who did not work in agriculture (0.6%; 95% CI, 0.6–0.7%; *p* = 0.001). Children who lived in homes that showed signs of rodents had significantly higher Th2 (0.8%; 95% CI, 0.6–0.7%) than children who did not (0.6%; 95% CI, 0.6–0.7%; *p* = 0.04). No significant difference was seen comparing children of women who held other jobs in agriculture with those who did not work in agriculture. Presence of a gas stove in the home also was associated with increased Th2 (0.7%; 95% CI, 0.7–0.8% vs. 0.5%; 95% CI, 0.4–0.6%; *p* = 0.002).

Having a mother working in agriculture was also associated with decreased levels of CD4, although this difference was not statistically significant when maternal work was further divided into fieldwork and other agricultural work. No other exposures were associated with Th1, Th2, or CD4 in bivariate analyses.

### Multiple linear regression models

The results of the regression analyses are presented in Table 3. In the final model for Th1 (adjusted *R*^2^ = 0.06), exclusive breast-feeding at 1 month of age (compared with partial or no breast-feeding) was associated with a 35.3% increase in Th1 (95% CI, 8.7–68.3%; *p* < 0.01) and owning pets was associated with a 34.5% increase in Th1 (95% CI, 6.3–70.1%; *p* = 0.01). Living below the poverty level, having signs of mold in the home, exposure to tobacco smoke, and mother working in the fields were also positively associated with Th1, but were not statistically significant. Breast-feeding status at 3 months of age was not associated with Th1 levels.

In the final model for Th2 (adjusted *R*^2^ = 0.09), mother working in the fields was associated with a 25.9% (95% CI, 0.8–57.3%; *p* = 0.04) increase in children’s Th2 levels and the presence of a gas stove in the home with a 46.8% (95% CI, 11.9–92.5%; *p* < 0.01) increase in Th2 at 24 months. Signs of rodents in the home and exclusive breast-feeding at 1 month were also associated with increased Th2, but not significantly. Results for Th1 and Th2 regression models were similar when CD4 levels were included in the model, suggesting that the associations with Th1 and Th2 were independent of the level of CD4 cells. In the final model for CD4 (adjusted *R*^2^ = 0.01), only the variable “mother working in fields” was marginally associated with a 2% decrease in children’s CD4 levels (*p* = 0.07).

## Discussion

In this study, the relationships between several environmental exposures during the first 12 months of life and levels of Th1 and Th2 cytokines were investigated in 239 24-month-old children living in an agricultural community. We observed a significant association between an elevated percentage of Th2 cells in children at 24 months of age and both physician-diagnosed asthma and maternal report of wheezing. This finding is consistent with the well-defined role of Th2 cytokines in the pathogenesis of asthma (Cohn et al. 2004; Larche et al. 2003; Romagnani 1994; Sanchez-Guerrero et al. 1997) and demonstrates that our flow cytometric method for identification of Th2 cells is suitable for use in epidemiologic studies.

We also found that exclusive breast-feeding at 1 month of age and ownership of pets were both significantly associated with higher percentage of Th1 in children at 24 months of age. Th2 was higher in children with mothers who performed agricultural fieldwork or had other agricultural workers living in the household. The presence of a gas stove in the home was also strongly associated with higher Th2, whereas a somewhat weaker relationship was seen between signs of rodents in the home and higher Th2. No important associations were observed with the percentage of CD4 cells.

Our results are consistent with other studies that have found associations between exposures to OP pesticides and self-reported exacerbation or onset of respiratory disorders in adults (Hoppin et al. 2002; Zhang et al. 2002) and children (Salam et al. 2004). Salam et al. (2004) found that exposure to herbicides and pesticides, in the first year of life, was associated with higher odds of athma in children. We observed that increased Th2 in children was associated with both the report of maternal work in agriculture and the presence of other agricultural workers in the child’s home. Although we cannot rule out the possibility that agricultural field work status also is a surrogate for other exposures, such as dust, coarse particulate matter, endotoxin, and other microbial products that are part of the agricultural environment, this association suggests that pesticide exposure may affect immune development, because agricultural work could be an indicator of pesticide exposure, and thus an indirect assessment of children’s exposure to pesticides.

The association between the report of a gas stove in the home and increased Th2 cells is consistent with other published literature indicating interactions between inhaled nitrogen dioxide and allergic responses. Controlled exposure to NO_2_ has been shown to enhance responses to inhaled allergen (Devalia et al. 1994; Krishna and Holgate 1999; Strand et al. 1998; Tunnicliffe et al. 1994). In one study, healthy, nonasthmatic and nonatopic adults were exposed for 4 hr to 2 ppm NO_2_ or filtered air for 4 consecutive days; subsequent analysis of samples of their bronchial epithelium showed that Th2 cytokines, including IL-5 and IL-13, were upregulated (Pathmanathan et al. 2003). Exposure to NO_2_ has been linked to respiratory symptoms in infants at risk for asthma (Garrett et al. 1998; van Strien et al. 2004), exacerbation of viral-induced asthma in children (Chauhan and Johnston 2003), and at least one report of an association between children’s personal exposure to NO_2_ and the onset of asthma (Infante-Rivard 1993). Another recent study of respiratory symptoms in the first year of life among 768 infants at above-average risk of developing asthma found that elevated NO_2_ levels were significantly associated with frequency of maternal reports of wheeze, persistent cough, and shortness of breath (Garrett et al. 1998; van Strien et al. 2004).

Breast-feeding has been reported to be associated with decreased risk of childhood asthma in most (Kull et al. 2004; Oddy et al. 2002), but not all (Rust et al. 2001; Wright et al. 2001) studies. We found that exclusive breast-feeding at 1 month of age was associated with significantly elevated Th1 in children at 24 months of age. It has been suggested that factors found in breast milk, such as soluble CD14, a receptor for bacterial lipopolysaccharide (LPS), is important in the prevention of childhood asthma (Rothenbacher et al. 2005) and atopy (Jones et al. 2002). CD14 transfers LPS to the Toll-like receptor-4 (TLR-4) expressed on a variety of cells in the immune system. The downstream consequence of TLR-4 signaling is IL-12 cytokine secretion (Palsson-McDermott and O’Neill 2004), which is a strong promoter of Th1 development. Because Th1 is considered to protect against development of asthma, our finding that exclusive breast-feeding is associated with increased Th1 levels could provide a mechanistic explanation of the observed associations between breast-feeding and the decreased occurrence of asthma.

We found that pet ownership during early childhood was associated with Th1 but not Th2, suggesting that pet ownership may be protective against the development of asthma in our cohort. Several studies have reported that exposure to pets (cats and dogs) in the first year of life can reduce the risk of allergic sensitization later in life (Holscher et al. 2002; Ownby et al. 2002; Roponen et al. 2005). One explanation that has been suggested is that pets, particularly dogs, are carriers of endotoxin (Gereda et al. 2000; Heinrich et al. 2001; Park et al. 2001) and that exposure to endotoxin is responsible for the Th1 bias. Our future studies will examine whether the increased Th1 status in this population is indeed attributable to household endotoxin (Gereda et al. 2000; Park et al. 2001; Roponen et al. 2005) by quantifying the endotoxin levels in house dust samples.

Previous studies have examined early environmental exposures in relation to the development of asthma and other allergic outcomes in children using mainly questionnaire responses about self-reported or physician-diagnosed asthma (Oddy et al. 2002; von Mutius et al. 2000; Wright et al. 2001). Our study is one of the first to associate these exposures with children’s Th1/Th2 levels, by flow cytometric detection of IFN-γ/IL-4 in T-helper cells in children’s whole blood. This method enumerates the number of memory cells that have developed in response to antigenic exposures, because only memory cells are capable of synthesizing the IFN-γ and IL-4 cytokines of interest within the 4-hr *ex vivo* stimulation period used to examine the T-helper cells (Pala et al. 2000). By using these immunologic markers, which are mechanistically relevant to asthma development, we showed that asthma and wheeze outcomes in children at 24 months are associated with elevated Th2 status in children at an early age. Furthermore, we used these biomarkers to identify, at a molecular level, associations between early environmental exposures (including maternal agricultural fieldwork, breast-feeding, pets, and gas stoves) and children’s immune status at 24 months of age.

## Correction

The values in Figure 1B were incorrect in the manuscript originally published online; they have been corrected here.

## Figures and Tables

**Table 1:** Characteristics of CHAMACOS study population [no. (%)].^a^

Characteristic	With Th1/Th2 data (*n* = 239)	Without Th1/Th2 data (*n* = 175)	*p*-Value
Health outcomes (at 2 years of age)			
Doctor-diagnosed asthma			
Yes	23 (9.6)	9 (5.1)	
No	216 (90.4)	168 (94.9)	0.09
Maternal report of wheezing (without a cold)			
Yes	12 (5.1)	12 (6.8)	
No	222 (94.9)	165 (93.2)	0.48
Doctor-diagnosed eczema			
Yes	17 (7.3)	17 (9.7)	
No	217 (92.7)	158 (90.3)	0.40
Doctor-diagnosed bronchitis/bronchiolitis			
Yes	106 (44.5)	66 (37.7)	
No	132 (55.5)	109 (62.3)	0.17
Doctor-diagnosed pneumonia			
Yes	68 (28.7)	39 (22.0)	
No	169 (71.3)	138 (78.0)	0.13
Exposures (between 0 and 1 year of age)			
Pesticides used in home			
Yes	142 (60.4)	104 (63.0)	
No	93 (39.6)	61 (37.0)	0.60
Mother’s work status			
Did agricultural fieldwork	73 (30.5)	48 (27.0)	
Did other agricultural work	20 (8.4)	14 (7.9)	
Did nonagricultural work/did not work	146 (61.1)	116 (65.2)	0.69
Father worked in agriculture (field or other)			
Yes	151 (63.2)	93 (52.3)	
No	88 (36.8)	85 (47.8)	0.03*
Any agricultural workers living in home			
Yes	131 (54.8)	95 (53.4)	
No	108 (45.2)	83 (46.6)	0.77
Gas stove in home			
Yes	198 (82.9)	142 (79.8)	
No	41 (17.2)	36 (20.2)	0.42
Owned pets			
Yes	75 (31.7)	62 (37.4)	
No	162 (68.4)	104 (62.7)	0.23
Signs of cockroaches in home			
Yes	152 (67.6)	100 (62.9)	
No	73 (32.4)	59 (37.1)	0.34
Signs of rodents in home			
Yes	97 (43.1)	56 (35.2)	
No	128 (56.9)	103 (64.8)	0.12
Signs of moderate to extensive mold in home			
Yes	141 (62.7)	106 (66.7)	
No	84 (37.3)	53 (33.3)	0.42
Infant feeding at 1 month			
Exclusive breast-feeding	126 (53.2)	93 (56.0)	
Partial breast-feeding	81 (34.2)	49 (29.5)	
Not breast-feeding	30 (12.7)	24 (14.5)	0.60
Household income			
Below poverty level	166 (69.5)	109 (61.2)	
Above poverty level	73 (30.5)	69 (38.8)	0.08

a Some totals will be lower because not every subject responded to all questions asked during interview.

* Statistically significant (*p* < 0.05).

**Table 2:** Associations between health outcomes (at 2 years of age), early exposures (between birth and 1 year of age) and Th1, Th2, and CD4 levels at 24 months of age [mean (95% CI)].

Characteristic	Th1	Th2	CD4
Health outcomes (at 2 years of age)			
Doctor-diagnosed asthma			
Yes	3.0 (2.6–3.4)	1.0 (0.7–1.2)*	31.9 (28.4–35.4)
No	2.9 (2.3–3.7)	0.7 (0.6–0.7)	33.0 (32.0–34.0)
Maternal report of wheezing (without a cold)			
Yes	4.1 (2.7–6.3)	1.2 (0.8–1.8)*	32.9 (31.1–34.8)
No	2.9 (2.6–3.3)	0.7 (0.6–0.8)	32.8 (31.7–33.9)
Doctor-diagnosed eczema			
Yes	3.0 (2.2–4.1)	0.7 (0.6–1.0)	33.0 (32.0–34.1)
No	3.0 (2.6–3.4)	0.7 (0.6–0.8)	32.2 (29.6–34.8)
Doctor-diagnosed bronchitis/bronchiolitis			
Yes	2.8 (2.3–3.4)	0.6 (0.6–0.7)	32.1 (30.9–33.3)
No	3.1 (2.6–3.6)	0.7 (0.6–0.8)	33.8 (32.2–35.3)
Doctor-diagnosed pneumonia			
Yes	2.7 (2.1–3.5)	0.6 (0.5–0.8)	33.2 (31.0–35.4)
No	3.1 (2.7–3.5)	0.7 (0.6–0.8)	32.7 (31.7–33.7)
Exposures (between 0 and 1 year of age)			
Pesticides used in home			
Yes	2.9 (2.5–3.4)	0.7 (0.6–0.8)	32.5 (31.3–33.7)
No	3.0 (2.5–3.6)	0.7 (0.6–0.8)	33.4 (31.7–35.0)
Mother’s work status			
Did agricultural fieldwork	3.4 (2.7–4.1)	0.9 (0.7–1.0)**	31.2 (29.8–32.6)
Did other agricultural work	3.1 (2.2–4.2)	0.7 (0.4–1.0)	33.2 (29.5–36.8)
Did nonagricultural work/did not work	2.7 (2.4–3.2)	0.6 (0.6–0.7)	33.7 (32.4–35.0)
Father worked in agriculture			
Yes	3.2 (2.8–3.7)	0.7 (0.6–0.8)	32.4 (31.3–33.6)
No	2.6 (2.1–3.2)	0.6 (0.5–0.8)	33.7 (32.0–35.4)
Any agricultural workers living in home			
Yes	3.0 (2.5–3.5)	0.8 (0.7–0.9)*	32.1 (30.9–33.3)
No	2.9 (2.5–3.5)	0.6 (0.5–0.7)	33.8 (32.3–35.4)
Gas stove in home			
Yes	3.0 (2.7–3.4)	0.7 (0.7–0.8)*	32.9 (31.9–34.0)
No	2.6 (1.8–3.7)	0.5 (0.4–0.6)	32.7 (30.0–35.3)
Owned pets			
Yes	3.5 (3.0–4.1)*	0.7 (0.6–0.8)	32.8 (30.8–34.9)
No	2.7 (2.3–3.2)	0.7 (0.6–0.8)	32.9 (31.8–33.9)
Signs of cockroaches in home			
Yes	3.2 (2.8–3.6)	0.7 (0.6–0.8)	32.6 (31.4–33.8)
No	2.8 (2.3–3.4)	0.7 (0.6–0.8)	32.9 (31.2–34.7)
Signs of rodents in home			
Yes	3.3 (2.8–3.9)	0.8 (0.7–0.9)*	32.7 (31.1–34.2)
No	2.8 (2.4–3.3)	0.6 (0.6–0.7)	32.8 (31.5–34.1)
Signs of moderate to extensive mold in home			
Yes	3.3 (2.8–3.8)	0.7 (0.6–0.8)	32.9 (31.7–34.1)
No	2.7 (2.2–3.2)	0.7 (0.6–0.8)	32.5 (30.7–34.3)
Infant feeding at 1 month			
Exclusive breast-feeding	3.3 (2.9–3.9)#	0.8 (0.7–0.9)	33.1 (31.7–34.5)
Partial breast-feeding	2.7 (2.3–3.2)	0.7 (0.6–0.8)	32.8 (31.2–34.4)
Not breast-feeding	2.3 (1.4–3.7)	0.5 (0.4–0.8)	32.1 (29.4–34.7)
Household income			
Below poverty level	3.1 (2.7–3.6)	0.7 (0.6–0.8)	32.6 (31.4–33.7)
Above poverty level	2.6 (2.1–3.2)	0.7 (0.6–0.8)	33.6 (31.8–35.5)

* Statistically significant (*p* < 0.05).

** Statistically significant (*p* < 0.05) compared with mothers who did not do agricultural fieldwork.

# Statistically significant (*p* < 0.05) compared with children who were not breast-fed exclusively.

**Table 3:** Regression models^a^ for Th1, Th2, and CD4 levels at age 24 months of age.

	Percent change (95% CI)	*p*-Value
Th1 (final adjusted *R*^2^ = 0.06)		
Mother worked in fields	17.7 (−7.2 to 49.2)	0.18
Household with pets	34.5 (6.3 to 70.1)	0.01
Signs of moderate to extensive mold in home	21.6 (−2.8 to 51.9)	0.09
Exclusive breast-feeding at 1 month	35.3 (8.7 to 68.3)	0.01
Exposed to tobacco smoke	39.2 (−9.9 to 114.9)	0.14
Below poverty	24.1 (−2.0 to 57.2)	0.07
Th2 (final adjusted *R*^2^ = 0.09)		
Mother worked in fields	25.9 (0.8 to 57.3)	0.04
Agricultural workers living in home	17.5 (−4.2 to −44.3)	0.12
Gas stove in home	46.8 (11.9 to 92.5)	< 0.01
Signs of rodents in home	19.3 (−2.7 to 46.3)	0.09
Exclusive breast-feeding at 1 month	21.1 (−1.1 to 48.4)	0.06
CD4 (final adjusted *R*^2^ = 0.01)
Mother worked in fields	−2.0 (−4.1 to 0.1)	0.07

a The full regression models included the following variables: mother worked in fields, mother worked in other agriculture, father worked in agriculture, other agricultural workers in the home, pesticides used in home, presence of roaches in home, presence of rodents in home, signs of mold in home, presence of gas stove, exclusive breast-feeding, and level of poverty. Variables were eliminated in a step-wise, backward regression if *p* > 0.2.
